# Macrophage Migration Inhibitory Factor in Clinical Kidney Disease

**DOI:** 10.3389/fimmu.2016.00008

**Published:** 2016-01-26

**Authors:** Annette Bruchfeld, Mårten Wendt, Edmund J. Miller

**Affiliations:** ^1^Department of Renal Medicine, Clinical Science Intervention and Technology (CLINTEC), Karolinska Institutet, Karolinska University Hospital, Stockholm, Sweden; ^2^Feinstein Institute for Medical Research, Manhasset, NY, USA; ^3^Hofstra University School of Medicine, Hempstead, NY, USA

**Keywords:** MIF, AKI, CKD, glomerulonephritis, vasculitis, MIF gene polymorphism, diabetic nephropathy, ADPKD

## Abstract

Macrophage migration inhibitory factor (MIF) is a proinflammatory cytokine implicated in acute and chronic inflammatory conditions, including sepsis, autoimmune disease, atherogenesis, plaque instability, and pulmonary arterial hypertension. MIF in plasma and urine is significantly elevated in patients with acute kidney injury (AKI) and elevated MIF in serum is associated with markers of oxidative stress, endothelial dysfunction, arterial stiffness, and markers of myocardial damage in chronic kidney disease (CKD). Furthermore, MIF seems to be involved in vascular processes and cardiovascular disease associated with CKD, glomerulonephritis, autosomal dominant polycystic kidney disease, and possibly also in progression to renal failure. Moreover, in active anti-neutrophil cytoplasmatic antibody-associated vasculitis, plasma MIF levels have been shown to be significantly elevated as compared with samples from patients in remission. A significant difference in the genotype frequency of high production MIF -173 G/C genotype has been found in end-stage renal disease, compared to controls. Inhibition of MIF in a diabetic nephropathy model ameliorated blood glucose and albuminuria and in a model of adult polycystic kidney disease cyst growth was delayed. Preclinical studies support a potential therapeutic role for MIF in AKI and in a number of CKDs, whereas these data in human disease are still observational. Future interventional studies are needed to delineate the role of MIF as a treatment target in clinical kidney disease.

## Introduction

Macrophage migration inhibitory factor (MIF) was one of the first cytokines that was identified after being isolated from the supernatants of T-lymphocytes, and initially described as a soluble factor with macrophage migration-inhibiting properties (1–3). It has later been shown that MIF is produced by a number of other cells, such as monocytes, macrophages, granulocytes, endocrine cells, epithelial cells, and endothelial cells (4, 5).

Migration inhibitory factor is a pleiotropic upstream proinflammatory integral mediator of the innate immune system, stimulating the release of multiple cytokines, including tumor necrosis factor (TNF)-α, with CD 74 being a binding receptor promoting the recruitment of leukocytes into inflammatory sites in a chemokine-like fashion (6). Three-dimensional X-ray crystallography has revealed that the MIF molecule contains a hydrophobic pocket, which has been identified as the proinflammatory active site of MIF (7) and compounds binding to this region decrease downstream MIF signaling (8, 9).

Migration inhibitory factor has been implicated in the pathogenesis of sepsis, autoimmune diseases, such as rheumatoid arthritis, systemic lupus erythematosus, and cardiovascular disease (CVD) (6, 10–13). In atherosclerosis animal models, aortic inflammation was reduced, and neointimal plaques were stabilized after administration of anti-MIF antibody (14, 15).

Chronic kidney disease (CKD) is a state of chronic inflammation with major implications for morbidity and mortality driven by a significant increased risk for CVD (16, 17). Delineating the role of inflammatory markers in atherosclerotic and inflammatory disease in CKD is therefore of considerable interest. Whether MIF has an important role in this area is not well known. This article therefore aims at reviewing available data on the role of MIF in acute kidney injury (AKI), CKD, diabetic nephropathy, inflammatory kidney disease, and genetic aspects of MIF and kidney disease.

## MIF and AKI

Urinary MIF has previously been reported to be increased, and associated with the severity of renal injury, in human glomerulonephritis and has also been suggested as a potential biomarker for acute kidney damage in acute pyelonephritis (18, 19). Similar findings have been demonstrated in kidney transplantation (20). Augmented plasma levels of MIF seem to be an early and predictive event of AKI in septic patients admitted to the ICU (21). In preclinical models, MIF stimulates leukocyte chemotaxis as well as tissue infiltration of leukocytes and induces multiorgan damage affecting both lungs and kidneys (6, 22–24). In a recent paper by Stefaniak et al., it was shown that increased plasma levels of MIF in patients undergoing liver transplantation was significantly more predictive than serum creatinine for AKI and the need for renal replacement therapy postoperatively (25).

## MIF and CKD, Implications for Cardiovascular Disease

The prevalence of CKD worldwide is 10–12% and its incidence is even greater in the elderly (26, 27). Systemic low-grade inflammation is associated with loss of renal function, and the uremic phenotype is also linked to premature aging and accelerated atherosclerosis (28, 29). CVD is a major challenge in this patient population in which mortality rates due to CVD are about 10–20 times higher in dialysis patients than those of the general population (30). A number of proinflammatory factors have been investigated, such as C-reactive protein (CRP), interleukin-6 (IL-6), TNF, and high-mobility group box-1 protein (HMGB1), and increased circulating levels have in most cases been shown to be associated with poor outcome (22–34). It has however been suggested that retention by reduced cytokine excretion or degradation in the kidney, not only increased production, may play a role for the elevated cytokine levels (35).

We have previously shown that circulating serum levels of MIF are significantly elevated in CKD stage 3–5 patients (*n* = 257), compared with controls (*n* = 53) in a cross-sectional study (36). MIF levels were also associated positively with markers of oxidative stress and endothelial activation, such as 8-hydroxy-2-deoxyguanosine (8-OH-dG) levels and ICAM-1 levels, but not with inflammatory markers, such as CRP, IL-6, and TNF. However, in contrast to most previously described cytokines, we observed no correlation between MIF and glomerular filtration rate (GFR).

Rammos et al. recently showed that plasma MIF levels correlated negatively with endothelial function by flow-mediated dilation of the brachial artery, and positively with arterial stiffness indices using applanation tonometry in patients with end-stage renal disease (ESRD). In a multivariate regression model, MIF was an independent predictor for arterial stiffness. A correlation between high MIF and high-sensitive troponin I also suggested an association with myocardial injury in these patients (37). Taken together, these studies indicate that MIF may play a role in vascular disease associated with CKD, but further studies are needed.

## MIF, Pulmonary Arterial Hypertension, and CKD

Pulmonary arterial hypertension (PAH) is characterized by endothelial dysfunction, vasoconstriction, and pulmonary vascular remodeling. Haddad et al. have shown that AKI is relatively common in individuals with PAH and is a strong predictor of early death (38). Furthermore, in the study of Shah et al. (39), the severity of CKD in patients with PAH was directly related to the risk of death.

Recent studies have shown clear links among MIF, PAH, and the concomitant pulmonary vasoconstriction and vascular remodeling (40, 41). The release of MIF and the vascular changes are due, at least in part, to increased oxidative stress (42). Our study in CKD stage 3–5 patients showed that elevated serum MIF concentrations are associated with markers of oxidative stress (plasma 8-OH-dG levels) and endothelial activation (ICAM-1) (36). This further suggests possible links between MIF in CKD and the associated pulmonary vascular changes and cardiac changes.

## MIF and Diabetic Kidney Disease

Diabetic nephropathy is one of the leading causes of CKD and dialysis dependency. Albuminuria and impaired GFR predominantly account for the increased mortality observed in type 2 diabetes (43). Accumulating evidence has revealed that immunological and inflammatory mechanisms may play a significant role in the development and progression of diabetic nephropathy, in addition to non-immunological factors (44, 45). Elevated MIF levels have been found in patients with impaired glucose tolerance and type 2 diabetes and have also been associated with coronary events in these patient populations (46–48). MIF protein expression and urinary MIF excretion, the latter preceding the onset of microalbuminuria, have been demonstrated in a diabetic mouse model (49). Glomerular and tubulointerstitial mRNA expressions of the MIF receptor CD74 were shown to be increased in Pima Indians with type 2 diabetes and diabetic nephropathy (50). As shown by Wang et al., treatment of the diabetic db/db mice with the MIF inhibitor ISO-1 significantly decreased blood glucose levels and albuminuria in these mice, suggesting that MIF inhibition may be a potential therapeutic strategy in diabetic nephropathy (51).

## MIF and Glomerulonephritis

It has been established that upregulation of renal MIF mRNA expression in the endothelium, glomerular, and tubular epithelial cells is closely related to macrophage accumulation and renal tissue lesions in experimental glomerulonephritis. By contrast, in the normal kidney, MIF mRNA and protein are largely restricted to tubular epithelial cells and some glomerular visceral and parietal epithelial cells (52). By using a neutralizing anti-MIF antibody, it was possible to partially reverse established crescentic glomerulonephritis (53). In human disease, a marked increase in both glomerular and tubular MIF mRNA and protein expression has been demonstrated in proliferative forms of GN, correlating with leukocyte infiltration, histologic damage, and renal function impairment (54). Elevated MIF concentrations have been measured in peripheral blood T cells and monocytes from patients with IgA nephropathy (IgAN), which is the most common form of primary glomerulonephritis and is characterized by IgAN immune complexes in the glomerular mesangium, proliferation of mesangial cells, infiltration of inflammatory cells, and progressive glomerular injury. MIF overproduction was also correlated with the intensity of acute exacerbation in these patients (55, 56). Polymeric IgAN isolated from patients with IgAN was able to induce MIF production in human mesangial cells, and anti-MIF treatment was shown ameliorate kidney injury and reduce glomerular TGF-β 1 expression in an experimental model of IgAN (57, 58).

## MIF and Vasculitis

Granulomatosis with polyangiitis (GPA) and microscopic polyangiitis (MPA) are diseases characterized by systemic small vessel necrotizing inflammation, commonly affecting the kidneys and with a close association with the presence of anti-neutrophil cytoplasmatic antibodies (ANCAs), thus known as ANCA-associated vasculitides (AAV). A number of inflammatory cells and proinflammatory mediators have been implicated in the pathogenesis, which is believed to be initiated by priming of neutrophils and monocytes in the circulation by inflammatory stimuli (59). Increased levels of circulating MIF in serum have been found particularly in active AAV patients, but not in patients with other forms of vasculitis, such as giant cell arteritis and polyarteritis nodosa (60). We studied MIF in a prospective study of incident AAV patients at induction treatment, and at follow-up at 3 and 6 months and found elevated plasma levels at baseline. MIF decreased significantly during follow-up when most patients were in remission, but remained still elevated as compared with controls at all time points (61). MIF levels did not correlate with CRP, creatinine, or organ involvement. MIF has intriguingly been shown to be induced from macrophages by low concentrations of glucocorticoids (GC), possibly acting as a counter-regulator for glucocorticoid action (62). However, we found no correlation between MIF levels and GC exposure possibly due to the unphysiologically high GC treatment doses at the time of sampling. Finally, since there is a known association between thyroid disease and AAV in addition to antithyroid drugs being associated with the development of ANCA and vasculitis (63, 64), we investigated the role of thyroid hormone activity and MIF in AAV. The thyroid hormone thyroxine (T4) and its dextrorotatory isomer (dextrothyroxine; d-T4), but not triiodothyronine (T3), bind to the hydrophobic pocket within the MIF molecule and has been shown to be a potent inhibitor of the inflammatory activity of MIF in a dose-dependent manner, which was clearly demonstrated by administration of exogenous d-T4 to mice with severe sepsis (65). Administration of the hormonally inactive d-T4 significantly improved survival, even in mice that had previously undergone thyroidectomy. In patients with severe sepsis, low plasma T4 concentrations were inversely correlated with plasma MIF concentrations (65). In our human study, there was a strong correlation over time between the baseline MIF/T4 ratio and the MIF/T4 ratio at 6 months in AAV patients in remission (61). Both the preclinical and clinical data therefore suggest that blocking the inflammatory active site of MIF may both reduce inflammatory responses and improve the availability of T4.

## MIF and Autosomal Dominant Polycystic Kidney Disease

Autosomal dominant polycystic kidney disease (ADPKD) is an inherited autosomal dominant disease characterized by renal cyst formation and is associated with renal interstitial inflammation and fibrosis. The most common is the PKD1 mutation that encodes for polycystin-1 (PC1), which frequently leads to loss of kidney function in mid-life (66). It was recently shown that MIF regulates cyst growth in a murine ADPKD model through several mechanisms and is also accumulated in cyst fluid of human ADPKD kidneys (67). In polycystin-1-deficient mice, recruitment and retention of renal macrophages were dependent on MIF, which promoted cyst expansion. By deleting MIF or by pharmacological inhibition, cyst growth was delayed in murine ADPKD models. Macrophage recruitment was associated with the upregulation of monocyte chemotactic protein 1 (MCP-1) and inflammatory cytokine TNF-α, which further induced MIF affecting renal epithelial cells and cyst development (68). These findings may potentially be very important as the therapeutic options in slowing ADPKD progression until recently have been limited. New drugs, such as tolvaptan, a vasopressin V(2)-receptor antagonist, have recently been approved after demonstrating an effect on the increased rate of total kidney volume and slowing down renal function decline. However, the long-term effect of tolvaptan is unclear and side effects may limit its use (68).

## MIF and Genetics

Migration inhibitory factor corresponding gene has known polymorphisms in the -794 CATT_(5–8)_ repeat and the single-nucleotide polymorphism (SNP) -173*G/C and is associated with increased susceptibility and severity of a number of inflammatory and autoimmune conditions (69, 70).

The frequency of high-producer MIF -173 G/C genotype was higher (10.1%) in ESRD than in controls (1.2%), suggesting that it may play a role in progression to renal failure. However, there was no clear association between the MIF genotype and type of kidney disease in ESRD (71). In children with idiopathic nephrotic syndrome, the high-producer MIF -173*C allele was significantly more common than in controls. Furthermore, this allele was more common in steroid-resistance cases and was also associated with significantly higher probability of ESRD compared with G/G homozygous patients within 5 years from onset (72). In a recent study, it was shown that patients with GPA have an increased frequency of high-expression MIF CATT, and higher plasma MIF levels. In a murine model of granulomatous vasculitis, higher MIF expression increased mortality and pulmonary granulomas while injection of anti-MIF mAb protected mice from dying suggesting a role for MIF in the pathogenesis of GPA (73).

## Conclusion

**Figure 1:**
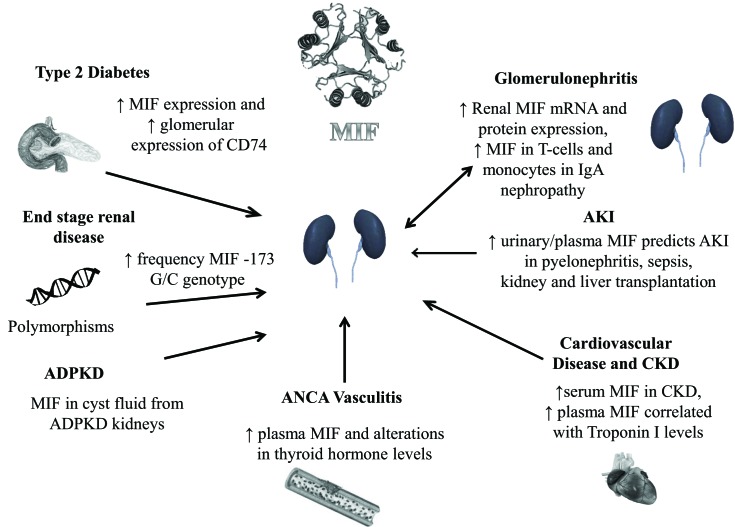
**MIF and human kidney disease**. Autosomal dominant polycystic kidney disease (ADPKD), acute kidney injury (AKI), chronic kidney disease (CKD).

We have here described the current evidence of MIF being a mediator in a number of diseases and conditions associated with kidney disease (Figure 1). Both MIF and its receptor CD74 may be potential biomarkers in these disorders and possible targets for pharmacological modulation. However, since MIF is also constitutively expressed it may be problematical to interfere with MIF activity in an interventional setting. Also, while we have discussed the possible detrimental effects of MIF, especially those associated with its inflammatory active site, the molecule can also be protective in certain circumstances. In particular, in addition to the inflammatory site, MIF also has an intrinsic thiol protein oxidoreductase activity. We, and others, have shown that this enzyme activity is protective against oxidative injury induced by ischemia reperfusion injury (74, 75).

Recently, Thiele et al. demonstrated that there are two redox-dependent conformational MIF isoforms. Oxidized MIF (oxMIF) is selectively expressed in the plasma and on the cell surface of immune cells of patients with different inflammatory diseases, but not in healthy individuals and is specifically recognized by three monoclonal antibodies (mAbs) directed against MIF. The authors also found a clear correlation between disease severity and the oxMIF/Cr ratio in the urine in patients with acute lupus nephritis, but not in patients with SLE without renal manifestations or in remission. Anti-oxMIF mAbs alleviated disease severity in a rat model of crescentic glomerulonephritis, interestingly with further improvement in synergy with GC (76). However, while there is much evidence, from preclinical studies, for the participatory role of MIF in the pathogenesis if several conditions few clinical trials of anti-MIF agents have been recorded. Anti-MIF mAb therapy is currently in phase I trials both for solid tumors (NCT01765790) and for lupus nephritis (NCT01541670). Whether inhibition of MIF or oxMIF may offer promising therapies in clinical conditions, such as AKI, CKD, diabetic nephropathy, inflammatory kidney diseases, and ADPKD, needs to be elaborated in future interventional studies.

## Conflict of Interest Statement

The authors declare that the research was conducted in the absence of any commercial or financial relationships that could be construed as a potential conflict of interest.
